# Validation of the basic need satisfaction for sport scale in Ethiopian athletes

**DOI:** 10.3389/fspor.2024.1424151

**Published:** 2024-08-22

**Authors:** Getabirhan Getinet Melesse, Zelalem Melkamu Tegegne, Sangeeta Rani

**Affiliations:** ^1^ Sport Science Department, Sport Academy, Bahir Dar University, Bahir Dar, Ethiopia; ^2^ Maa Omwati College of Education, University/College-MOIEC, Palwal, India

**Keywords:** internal consistency, psychological needs, reliability, self-determination theory, validity

## Abstract

By anchoring on the self-determination theory in an Ethiopian context, this study tried to establish the basic need satisfaction sport scales (BNSSS) reliability and validity. Despite the scale's usefulness in measuring athletes' psychological need fulfillment during a sporting event, no study has proven the scale's validity in a setting of Ethiopian sports. To validate the BNSSS scale, confirmatory factor analysis was used in the study. The 20 items of the BNSSS questionnaire's English translation are divided into five categories: relatedness, competence, autonomy-perceived locus of internal causality, autonomy-choice, and volition. Senior language experts translated the BNSSS questionnaire into Amharic. The Amharic version of the instrument was used to gather data from 321 athletes, 174 men, and 147 women, with a mean age of 23.34 22.59 and a standard deviation of 5.08 and mean age 5.32; a standard deviation of 2.33 year of experience in their sports from four baseball games. With a Cronbach's alpha value ranging from 0.848 to 0.882 (IPLOC to Volition respectively) across the five subscales and, the results confirm the reliability of the BNSSS for evaluating satisfaction with basic needs and motivation among Ethiopian athletes.” The result demonstrated an acceptable fit with the data (CFI, = 0.958, GFI, = 0.933, RMR, = 0.76, RMSEA, = 0.39) as well as internal consistency. All of the components' Cronbach's alpha values met expectations. The instrument's Amharic translation was thus valid and reliable for determining the extent to which Ethiopian athletes' basic needs were met.

## Introduction

A growing body of research studies has recognized basic psychological needs as the key factors that determine and sustain the optimum performance of athletes (1–3). These studies indicated that athletes' sports performance depends on many variables. Almagro et al. (4) indicated that among the influential variables affect sports performance include the three basic psychological needs whose satisfaction affects their motivation to perform better, continue participating in sports activities, and win the competition.

A self-determination perspective contends that variations in motivational levels rely on whether or not athletes are able to satisfy their demands in terms of the three basic psychological requirements (4). Because athletes need to feel engaged and motivated as members of the team, it is crucial to understand how they see their performance (5). In other words, along a linear continuum athletes' motivation levels increase as they gain an increasing degree of satisfaction with their three basic requirements (2). The more the three basic psychological demands are met by athletes, the more their athletic performance will increase (2).

Self-determination theory is an empirical macro theory of human motivation, psychological growth, and well-being (6). It can be used to explore social circumstances that support or deprive individuals of various sorts of motivation and fundamental psychological needs. According to this theoretical perspective, there are intrinsic and extrinsic motivated behaviors which are driven by the fulfillment of fundamental human needs such autonomy, competence, and relatedness (6). In recent studies examining how different levels of basic psychological needs affect the different types of motivation in the context of athletic sports performance is becoming more and more popular (7, 8). Basic psychological needs have been identified as the primary determinants of and maintainers of the peak athletic performance (1, 2). These studies found that various variables affect an athlete's sporting performance. According to (2), among the key factors that influence sports performance are the three basic psychological demands. In other words, personal fulfillment influences drive athletes to perform better, continue participating in sports activities and win the competition.

A self-determination perspective argues that competence, autonomy, and relatedness are the three essential psychological requirements (7) that an athlete must meet in order to perform well in sports. The degree to which athletes need social situations that encourage autonomy, competence, and relatedness may differ by culture despite the assumption that all people have the same fundamental psychological demands (9). For instance, the level of motivation emphasized by a coach of a regional national league football team may differ from another coach of the national football club. For a person to develop psychologically to its full potential, the basic psychological needs must be met (7).

Several studies have investigated the concept of basic psychological needs. For instance, Brien et al. (10) developed the Basic Psychological Needs at Work Scale (BPNWS), while Vlachopoulos and Michailidou (11) created the Basic Psychological Needs in Exercise Scale. The latter scale consists of 12 items divided into three constructs: competence, autonomy, and relatedness, with four items for each.

The BPNWS demonstrated good internal consistency, with Cronbach's alpha coefficients of 0.81 for competence, 0.84 for autonomy, and 0.92 for relatedness. The model also exhibited a good fit. Similarly Wilson et al. (12) developed a Psychological Need Satisfaction in Exercise Scale consisting of 18 items (six per dimension). This scale also showed high internal consistency, with Cronbach's alpha values of 0.91 for competence and autonomy, and 0.90 for relatedness. The model fit indices for this scale were also satisfactory.

Unlike the previously mentioned scales, which primarily focused on the health benefits of physical activity, Gillet et al. (13) developed the Echelle de satisfaction des besoins psychologiques to assess psychological needs in both physical exercise participants and athletes. This scale comprised 15 items, five for each need, and used a 7-point Likert scale ranging from “not true at all” to “very true.” The scale demonstrated acceptable internal consistency, with Cronbach's alpha values of 0.70 for competence, 0.82 for autonomy, and 0.81 for relatedness. Additionally, the model fit indices, CFI and IFI, were both excellent at 0.95, and RMSEA was acceptable at 0.06.

“While the previously mentioned scales demonstrated good reliability and validity in measurements, they did not specifically assess the satisfaction of basic psychological needs in elite athletes’ sports. Ng et al. (14) introduced a tool specifically designed to address this gap for measuring basic need satisfaction in competitive sporting contexts. This tool, called The Basic Need Satisfaction in Sport Scale (BNSSS), consists of 20 items: five for competence, five for relatedness, and ten for autonomy (divided into three dimensions: autonomy-choice (four items), autonomy-volition (three items), and autonomy-internal perceived locus of causality (IPLOC) (three items)). Ng et al. (14) applied the BNSSS to a sample of affiliated athletes. The internal consistency of the BNSSS yielded Cronbach's alpha coefficients of 0.77 for competence, 0.82 for autonomy-choice, 0.61 for autonomy-volition, 0.76 for autonomy IPLOC, and 0.87 for relatedness. The model fit indicators also showed favourable results: NNFI = 0.96, CFI = 0.97, RMSEA = 0.06, and standardized root mean square residual (SRMR) = 0.07.”

The exceptional performance of Ethiopian distance runners is often attributed to a combination of factors including genetic makeup, early exposure to running, physiological adaptations to altitude, diet, and strong economic motivation. These athletes have dominated middle and long-distance events Wilber et al. (15). While these factors have been well-studied and contribute to success in athletics, their relevance to other sports, such as ball games, is less clear due to a scarcity of research in our specific cultural and geographical context. To bridge this knowledge gap and understand the potential of Ethiopian athletes in different sports, it is crucial to explore factors like basic psychological needs, which have been relatively unexplored in this context (15).

It is observed the lack of questionnaire translated and validated to Ethiopian (Amharic language) to useful with Ethiopian athletes. In this sense, the BNSSS has been an adequate instrument to evaluate athlete's psychological needs in sports setting, however it has not been translated and validated in Ethiopian athletes. So the main aim of this present study was to translate and validate the BNSSS in to Amharic (an Ethiopian language) through adequate translation guide lines, to examine the factor structure, translation and adaptation of the original instrument, an invariance analysis, composite reliability, average variance extracted and to assess the reliability through internal consistency.

## Research methods

### Participants

The sample comprised of 321 athletes from various team sports, (*n* = 174, 54.21% males, and *n* = 147, 45.79% females) selected through purposive sampling from different sports federations within Ethiopia. Participants in the study ranged in age from 18 to 32 (M: 22.59; SD: 3.29). All of the athletes who took part in this study were members of their respective sport federations and had been actively participating in their chosen sport for at least two years on average (M = 5.32 years, SD = 2.33) and range of 2–12 years of experience. The participants’ demographic charactristics stated in Table 1. During the permission process, the participants were informed of the purpose of the study and their ethical rights; their responses are held in confidence. The correlation between the five BNSSS construct has been studied as shown in Table 2. Strong correlation has shown between internal perceived locus of causality and relatedness 0.631^**^ and weak correlation observed between competence and relatedness 0.108^*^.

**Table 1:** Demographic characteristics.

Sport type	Gender		T	MM_age_	MSD_age_	MM_exp_	MSD_exp_	WM_age_	WSD_age_	WM_exp_	WSD_exp_	TM_age_	TSD_age_	TM_exp_	TSD_exp_
	M	W													
FB	59	54	113	21.4	3.05	4.9	1.96	20.7	2.35	3.94	1.83	22.7	2.34	3.94	1.82
VB	46	38	84	24.4	2.75	6.09	2.15	23.76	2.98	6.03	2.33	24.08	2.85	6.06	1.02
BB	36	29	65	22.7	3.44	5.08	2.23	22.41	2.45	5.55	2.16	22.57	3.02	5.29	2.19
HB	33	26	59	25.3	3.86	7.24	2.64	20.85	1.89	4.04	1.18	23.36	3.85	5.83	2.65
Men			174	23.2	3.5	5.72	2.4								
Women			147					21.9	2.8	4.8	2.2				
Total	174	147	321									22.6	3.3	5.3	2.3

MM_age_, Men mean age; SD, standard deviation; exp, experience; T, total; W, women.

**Table 2:** Correlations between study variables.

Dimensions	COP	AIPLOC	VOLI	CHO	REL
COP	1.000	.128*	.113*	.153**	.108*
AIPLOC	.128*	1.000	.338**	.543**	.631**
VOLI	.113*	.338**	1.000	.209**	.366**
CHO	.153**	.543**	.209**	1.000	.571**
REL	.108*	.631**	.366**	.571**	1.000

COP, competence; AIPLOC, autonomy IPLOC; VOLI, volition; CHO, choice; and REL, relatedness.

* Correlation is significant at the 0.05 level (1-tailed).

** Correlation is significant at the 0.01 level (1-tailed).

### Basic need satisfaction sport scale (BNSSS)

The basic psychological need satisfaction of individuals is assessed by Basic Psychological Needs Satisfaction Sport Scale (BNSSS) instrument, developed by (14). It was the one of preferred measurement sport specific tool which showed valid psychometric properties (16). The BNSSS comprises of 20 measures that fall into five categories of: relatedness, competence, autonomy choice, autonomy volition, and autonomy (IPLOC) internal perceived locus of causality. The BNSSS is an inclusive instrument that uses a seven-point likert-type answer scale with responses ranging from 1 (“not true at all”) to 7 (“very true”) higher scores represent greater psychological need fulfillment. Ng et al. (14) demonstrated that BNSSS have adequate internal consistency scores alpha coefficient of: 0.77 for competence; 0.82 for autonomy-choice; 0.76 for autonomy-IPLOC; 0.61 for autonomy-volition, and 0.77 for relatedness constructs. The current study also yielded adequate internal consistency Table 3 scores for competence (*α* = 0.88), autonomy-choice (*α* = 0.879), autonomy-IPLOC (*α* = 0.85), autonomy-volition (*α* = 0.882), relatedness (*α* = 0.869) and Cornbrash's alpha of 20 item BNSSS is 0.858 while Mc Donalds Omega value of 20 items BNSSS is 0.868 see Table 4. The current study also calculated its average variance extracted and composite reliability for each sbscales for assuring adequate discriminant and convergent validity can be viewed in Table 3. The BNSSS has a good factor structure of (CFI = 0.958; RMSEA = 0.053), indicating that the instruments validity is good. As a result, the present translation of BNSSS into Amharic study examined the reliability and validity of the Amharic translation using internal consistence and construct validity to ensure that it can be used to measure Ethiopian athletes satisfaction with their basic needs.

**Table 3:** Descriptive statistics and intra-class coefficients of BNSSS subscale scores.

Subscales	Test					Re-test					
	*α*	M	SD	CR	AVE	*α*	M	SD	CR	AVE	ICC
Competence	.880	559	.71	.88	.59	.93	551	.82	.92	.724	.929
Volition	.882	60	.84	.72	.88	.91	597	.89	.908	.769	.896
Choice	.879	52	12	.88	.65	.97	49	14	.973	.903	.973
Iploc	.848	61	.85	.85	.65	.94	59	11	.940	.841	.931
Relatedness	.869	52	114	.56	.87	.96	534	112	.956	.814	.954

*α*, Alpha; M, Mean; SD, standard deviation; CR, composite reliability; AVE, average variance extracted; ICC, intra-class correlation.

**Table 4:** Frequentist statistics scale reliability of men and women BNSSS *N* = 321.

Estimate	McDonald's *ω*	Cronbach's *α*
Point estimate	0.868	0.858
95% CI lower bound	0.847	0.836
95% CI upper bound	0.889	0.879

### Instrumentation

Ethiopian athletes took part in the study. Participants' responses were gathered using an Amharic version of the recent version of the Basic Psychological Need Satisfaction for Sport Scale (BPNSS) Questionnaire. The basic psychological needs satisfaction for sport scale surveys with 20 items was completed by the participants. The 20 questions that made up this sport scale are divided into five subscales. This study established the validity and reliability of the Amharic (an Ethiopian language) translation and validation of the basic psychological needs satisfaction for Sport Scale (BPNSS) Questionnaire in English. All subscales Cronbach's internal reliability coefficients were excellent.

### Procedures

First, Bahir Dar university sports academy's ethics commission approved the research project: the validation of BNSSS's use with Ethiopian athletes, followed by by research in to relationship among motivation in sport and sports performance. The leading author then met with the team captains and head coaches to discuss the purpose of the study, presenting them with a letter from the sports academy dean's office asking for their voluntary cooperation during the data collection process. Then permission was granted to collect data directly from the participants. In order to ensure that the responses provided by the athletes were more informative of their psychological states, questionnaires were issued to the participants during the competitive season. Four data collectors distributed the BNSSS questionnaire to the athletes after briefing the overall purpose of the data collection. To translate and adapt the BNSSS instrument from its original English language version, first developed by (14) to Amharic' the methodological procedures recommended by Vallerand (17) and endorsed by Banville et al. (18) was followed:

1. An initial translation with the assistance of three translators who were proficient in the English and Amharic language;
2. Evaluation of the initial Amharic version by four experts independently;
3. Four additional experts collectively examinedall the items until they reached a consensus on item wording;
4. We administered this version of the questionnaire to 81 (men = 45; women = 36) independent athletes from four team and three individual sport types to assess item clarity and accuracy (pilot study);
5. Two Amharic language experts conducted a final review of the Amharic version of the BNSSS to ensure correct syntax, spelling, and grammar were correct/(Final version).

### Data analysis

The data sets of 321 participants were first looked at for possible missing data and the verification checked for potential outliers. The skewness and kurtosis of the replies were used to evaluate the items' univariate normality Table 5. The statistical package for social sciences (SPSS) version 26.0 software was utilized to calculate the descriptive statistics Table 5 on a sample size of 321 data. Alpha coefficients were used to assess the internal consistency of sub scale (19).

**Table 5:** Descriptive statistics and item-factor loadings of BNSSS item scores and cronbach's alpha.

Dimension	Item number	M	SD	Skewness	Kurtosis	IFL	ET	*α*
Competence	6	5.55	.904	−.392	−.446	.72	.52	.880
	11	5.60	.879	.005	−.520	.76	.58	
	12	5.62	.873	−.117	−.253	.84	.71	
	14	5.45	.958	−.104	−.341	.74	.54	
	17	5.73	.812	−.442	−.035	.80	.64	
AIPLOC	2	5.99	1.012	−1.539	2.312	.72	.52	.848
	15	6.23	.894	−1.943	4.088	.83	.69	
	16	6.10	1.004	−1.467	1.328	.87	.66	
Autonomy volition	3	6.06	.967	−.947	.802	.91	.83	.882
	5	5.97	.925	−.774	.228	.82	.67	
	8	6.09	.909	−.789	−.190	.81	.66	
Autonomy choice	4	5.27	1.470	−1.567	2.018	.83	.69	.879
	9	5.44	1.524	−1.925	3.532	.78	.60	
	13	5.17	1.488	−1.018	.188	.84	.70	
	20	4.98	1.556	−.884	−.434	.78	.60	
Relatedness	1	5.20	1.512	−1.170	.295	.79	.63	.869
	7	5.40	1.319	−1.931	4.664	.72	.52	
	10	5.21	1.470	−1.348	.991	.74	.54	
	18	5.24	1.402	−1.300	1.816	.76	.57	
	19	5.45	1.334	−1.866	3.979	.78	.61	

M, Mean; SD, standard deviation; IFL, item-factor loadings; Et, error term.

A value of alpha coefficient above 0.7 is typically recommended and suggested threshold value and defined as acceptable for internal consistency (20–22). Furthermore, a criterion for retaining or removing items from the measurement model was a significant factor loading of greater than 0.5 with modification index.

Hair et al. (23) stated that all standardized factor loadings should be at least 0.5 and, ideally, at least 0.7.factor loading. Scale score analysis was carried out using confirmatory factor analysis (CFA). Using LISREL 8.5, CFAs were performed to confirm the factorial structure of the five dimensions of the Ethiopian version of the BNSSS. Only the desired construct was allowed to load the item scores. Error phrases were not allowed to correlate, but factors were. To assess model fit, a variety of goodness-of-fit indices were employed. They included the non-normed fit index (NNFI), comparative fit index (CFI), standardized root mean square error (SRMR), and root mean square approximation error (RMSEA). The chi-square (*x*^2 ^= 301.974, DF* *= 160), NNFI, and CFI were also among them. Values of NNFI, CFI > .90, and RMSEA.08 have historically been employed as indicators of an adequate fit (24). Hu and Bentler (25), who made this suggestion more recently, said that NNFI and CFI values of at least 0.95 were necessary for satisfactory model fit, whereas SRMR and RMSEA shouldn't go over 0.08 and 0.06, respectively. However, using these more difficult cutoff values could lead to more Type I mistakes (26). As a result (25), Criteria were employed to indicate very good fit while the old criteria were used to indicate good model fit. The item level mean scores ranged from 4.84 (SD: 1.85; autonomy choice item 20) to 6.17 (SD: .89; autonomy volition, item 8 represents lowest standard deviation value), according to the descriptive statistics for the BNSSS components. The range of SD was found to be between .84 for item 12 on competence to 1.82 for item 20 on autonomy and choice. In the CFA, the model was investigated using the maximum likelihood estimation technique.

## Results

**Figure 1:**
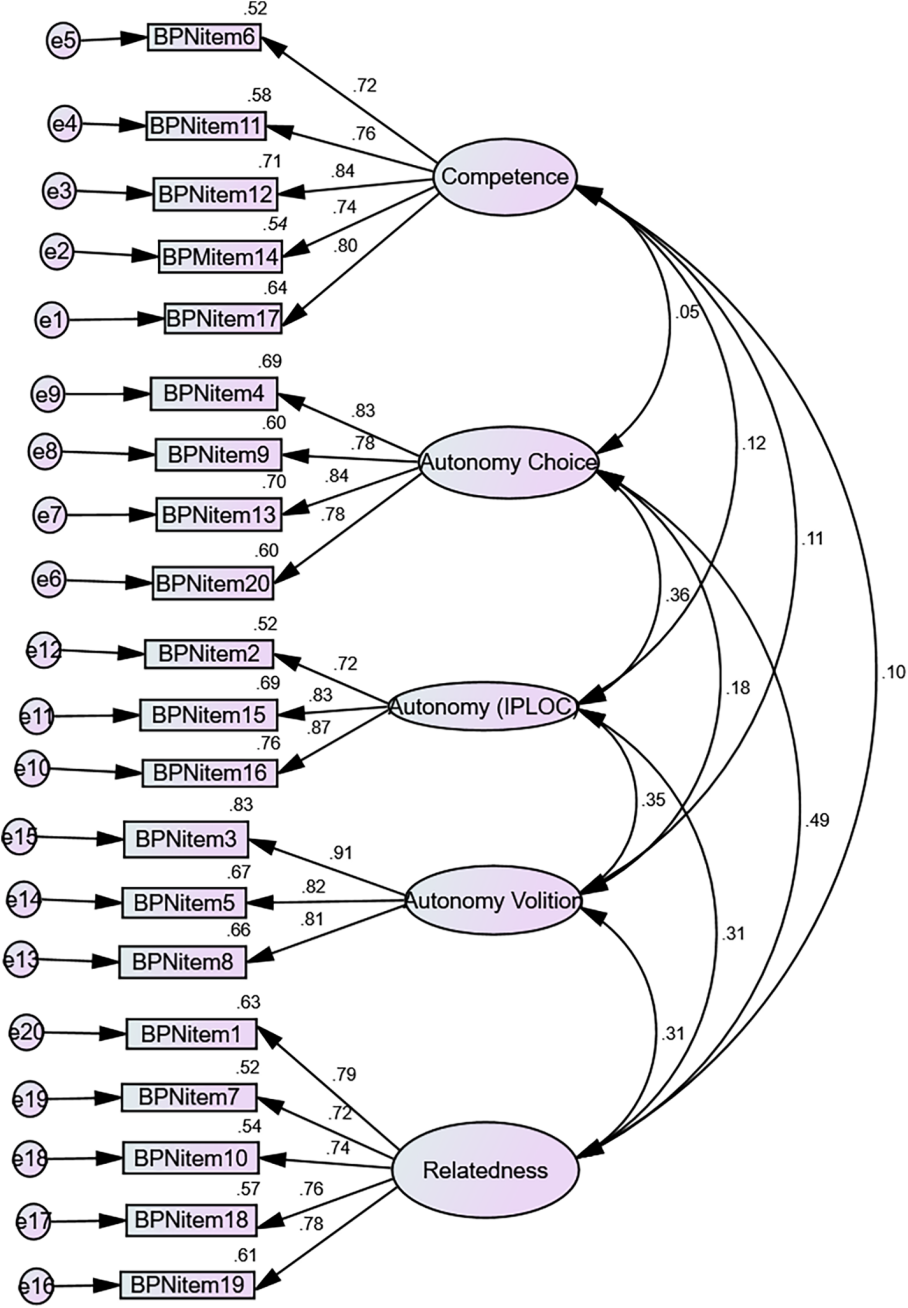
Factor structure of BNSSS item scores.

The data analyses showed that the single-factor model fits the data well Table 6 CMIN/DF. = 1.887, CFI = 0.958, TLI = 0.950, GFI = .93, IFI = 0.958, RMSEA = 0.053; RMR = .069; and SRMR = 0.469. Statistics showed that the standardized factor loadings were significant Figure 1.

**Table 6:** Fit indices for five model BNSSS.

Parameters	*X* ^2^	DF	*P*-value	*X*^2^/DF	GFI	RMSEA	TLI	NFI	CFI	IFI	SRMR	RMR
Values	301.97	160	<0.001	1.887	0.91	0.053	0.95	0.916	0.958	0.958	0.0469	0.069

A measurement model was first used to assess the later-presented structural equations model (SEM), which corresponded to a confirmatory factorial analysis (CFA) and allowed for the building of scale validity. “The cronbach's alpha values are 0.880 for competence, 0.848 for autonomy- internal perceived locus of causality, 0.882 for autonomy-volition, 0.879 for autonomy-choice, and 0.869 for relatedness, indicating high internal consistency both Cronbach's alpha and Mc Donalds Omega across all subscales Table 3 and Table 4.”

The floor and ceiling effect for BNSSS data in Table 7 showed that the scale is appropriately sensitive, the sample is representative and the distribution of scores reflects the actual distribution of basic need satisfaction among the population of interest. Additionally the data is reliable which is consistent scores across different measurement (27). In essence, a good floor and ceiling effect for BNSSS data suggests that the scale is working as intended and that the data collected is meaningful.

**Table 7:** The ceiling and floor effect of each BNSSS items.

Maximum and minimum percent of scores			
Dimensions	Items	Minimum	Maximum
Competence	Co 6	7.6%	13%
** **	Co 11	1.96%	10.3&
** **	Co 12	7.8%	14.8%
** **	Co 14	2.35%	11%
** **	Co 17	2%	11.5%
IPLOC	IP 2	2.7%	13.9%
** **	IP 15	2.9%	14.3%
** **	IP 16	2.19%	13.47%
Volition	Vol 3	1.897%	10.33%
** **	Vol 5	1.6%	12.7%
** **	Vol 8	2.36%	11.33%
Choice	Cho 4	1.92	9.46
** **	Cho 9	2.26	10.89
** **	Cho 13	1.93	12.59
** **	Cho 20	1.99	7.2
Relatedness	Rel 1	2.13	11.47
** **	Rel 7	1.56	9.15
** **	Rel 10	1.79	10.6
** **	Rel 18	2.12	11.4
** **	Rel 19	2.26	13.9

In Table 3 above the result indicated that an excellent value of AVE and CR exhibiting strong convergent and discriminant validity as well as high internal consistency. Acceptable AVE indicates that the latent variables explain a substantial amount of variance in their respective items suggesting good construct validity while the acceptable value of composite reliability suggest the items are highly correlated, indicating strong internal consistency and reliability of the construct. We plan to address this critical gap concurrent validity in future research by outline proposed methods, such as correlating the scale with other relevant measures or comparing scores between different groups.

## Discussion

BNSSS which is grounded to SDT is newly developed questionnaire for assessing athletes applied to sport competitive context (14). The current study was aimed at translating, adapting and validating the Ethiopian version BNSSS and its SDT framework.

After the translation process, EFA conducted to establish the construct validity of BNSSS Ethiopian version. The data from this study were better fit for the model.

“Research has continually highlighted the importance of psychological capacities, such as decentering ability, in modulating the impact of psychological conditions on sports performance (28). In the context of our study, the findings suggest that the validity of the BNSSS scale might be influenced by similar mediating or moderating factors, as explored in the moderated mediation model by Diotaiuti et al. (28). This perspective is crucial for understanding how athletes’ basic psychological needs can be better met, directly influencing their motivation and performance.”

The goal of this study was to translate, adapt, and test the reliability and validity of the Ethiopian version of the Basic Need Satisfaction Sport Scale Questionnaire for team sports. The 20-item, five-factor BNSSS was developed to assess satisfaction with basic psychological needs such as competence, autonomy, and relatedness (29), which is based on self-determination theory. The autonomy construct consists of ten items divided into three subcategories: choice (four items), IPLOC (three items), and volition (three items); the other construct of basic psychological needs, such as relatedness and competence, contains five items each. The subsequent CFA supported the five-factor BNSSS model (competence, relatedness, autonomy choice, autonomy volition, and autonomy internally perceived locus of causality (IPLOC).

The result indicated that the Ethiopian version showed adequate reliability and validity or replicated of the original and other subsequent version (7, 14, 30), for this sample. In terms of scale reliability, the subscales had acceptable internal consistency, with values comparable to those reported in the previous study. In terms of model fit, this study obtained a good fit for the original BNSSS with X2/DF = 1.887, CFI = 0.958, and RMSEA = 0.053. The findings align with previous research (14, 30).

The correlation between BNSSS subscales in Ethiopian version were higher than those in the original version studied by (14), suggesting that the translated scale may be particularly coherent in the context of Ethiopian sports.

The present study had some limitations. One of them was that it was limited to ball game athletes and had a sample size of 321; that is, the study excluded individual sports and very young athletes. As a result, it is recommended that it would be studied comprehensively in a diverse group of Ethiopian athletes with varying levels of athletic involvement. We also recommend as Ethiopia is multilingual country so as athletes speak different language, the questionnaire has to be translated accordingly. Additionally future studies can use interview session. Finally, as (14) stated, “Scale development” is “an ongoing” process, so it is a guarantee to researchers in the future to investigate the advancement of the scale.

## Conclusion

In conclusion, the findings of this current study, which investigated the reliability and validity of the Ethiopian version BNSSS, show that all five dimensions have high levels of validity and internal consistency. The study's main findings indicate that satisfaction levels with the three sub dimensions of autonomy, competence, and relatedness BPN in sport are high. Furthermore, a significant and positive correlation was found between the five basic psychological needs (BNSSS) constructs. It has been discovered that satisfaction with BPN is an excellent predictor of self-determination theory. The results emphasize the importance of satisfying BPN for enhancing athlete's motivation and performance. “In conclusion, the study validates the Amharic version of the BNSSS, confirming its reliability and validity for assessing the satisfaction of basic psychological needs among Ethiopian athletes. This supports the scales applicability in diverse cultural settings, there by extending its utility beyond the initial validation context,” We recommend using it to assess athlete satisfaction in various regions of the country, at various age levels, and in individual sports.

## Data Availability

The original contributions presented in the study are included in the article/Supplementary Material, further inquiries can be directed to the corresponding author.
